# Spherical Bezier Curve-Based 3D UAV Smooth Path Planning Utilizing an Efficient Improved Exponential-Trigonometric Optimization

**DOI:** 10.3390/biomimetics11020085

**Published:** 2026-01-23

**Authors:** Yitao Cao, Kang Chen, Gang Hu

**Affiliations:** 1 Unmanned System Research Institute, Northwestern Polytechnical University, Xi’an 710072, China; caoyit@163.com; 2 Department of Applied Mathematics, Xi’an University of Technology, Xi’an 710054, China

**Keywords:** path planning, unmanned aerial vehicle, exponential-trigonometric optimization, spherical Bezier curve, alpha evolution strategy, noise and physical attack strategy, opposition-based cross teaching strategy

## Abstract

Path planning, as a key technology in unmanned aerial vehicle (UAV) systems, affects the overall efficiency of task completion and is often limited by energy consumption, obstacles, and maneuverability in complex application environments. Traditional algorithms have insufficient performance in nonlinear, multimodal, and multiconstraints problems. Based on this, this paper proposes an improved exponential-trigonometric optimization (ETO) to solve a 3D smooth path planning model based on a spherical Bezier curve. Firstly, a fixed arc length resampling strategy is proposed to address the issue of the insufficient adaptability of existing path smoothing methods to dynamic threats. Generate a uniformly distributed set of reference points along the Bezier curve and combine it with spherical projection to improve the safety and efficiency of the flight path. On this basis, establish a total cost function that includes four types of costs. Secondly, a new ETO variant called IETO is proposed by introducing the alpha evolution strategy, noise and physical attack strategy, and opposition-based cross teaching strategy into ETO. Then, the effectiveness of IETO for addressing various optimization problems is showcased through population diversity analysis, ablation analysis, and benchmark experiments. Finally, the results of the simulation experiment indicate that IETO stably provides shorter and smoother safe paths for UAVs in three elevation maps with different terrain features.

## 1. Introduction

As an aircraft capable of autonomous flight, a UAV offers numerous advantages, including compact size, agile operation, cost-effectiveness, and high adaptability. In recent years, UAVs have shown excellent performance in both military and commercial fields, and often perform high-intensity tasks in complex and dangerous environments. In the military realm, UAVs are primarily used for reconnaissance and suicide attacks [1]. For example, UAVs carrying warheads can intelligently identify targets that need to be struck and approach them discreetly. In the commercial realm, the UAV is used for agricultural irrigation and logistics distribution [2]. With the application of various intelligent technologies in urban environments, UAV express delivery that can carry packages and automatically deliver them is becoming an emerging logistics method, and it has the advantages of improving overall operational efficiency and reducing labor cost [3]. In addition, UAVs play a crucial role in disaster response, enabling rescue teams to swiftly assess the situation and plan their actions [4]. However, UAVs may face various challenges during flight, including avoiding obstacles in the environment, maintaining communication links between UAVs, and minimizing energy consumption [5]. Whether the flight path of the UAV can meet various requirements in practical work will directly affect the efficiency of UAV task completion. Therefore, effective path planning algorithms are needed to design a safe and efficient flight trajectory for UAVs [6].

UAV path planning belongs to the NP-hard problems and is usually modeled as a discrete constrained optimization problem [7]. Scholars have classified UAV path planning methods into three categories based on principles, including traditional algorithms, reinforcement learning algorithms, and nature-inspired algorithms (NIA) [8]. Traditional algorithms can be divided into three categories. Firstly, the unit decomposition method treats the search space as a grid and discretizes the paths, including A* algorithm [9] and Dijkstra algorithm [10]. Secondly, the graph search method obtains a search tree containing feasible path points by randomly sampling the space, with representative algorithms including the RRT algorithm [11] and the PRM algorithm [12]. Due to insufficient consideration of UAV maneuverability, graph search method often generates dangerous paths that cause the UAV to deviate from its trajectory at turns [13]. Thirdly, APFM considers the workspace of the UAV as a potential field containing both gravitational and repulsive forces, and new APFM variants include MPAPFA [14] and CAPFA [15]. However, APFM can make it difficult for UAVs to quickly stabilize at the target point in environments with a large number and dense distribution of obstacles [16]. The feasible paths found by traditional algorithms often fail to achieve optimal solutions and are not suitable for complex nonlinear or multimodal problems. Reinforcement learning algorithms can quickly provide new optimal paths for UAVs when the environment undergoes dynamic changes, with representative algorithms including deep deterministic policy gradients [17] and the Q-learning algorithm [18]. However, reinforcement learning algorithms cannot guarantee the accuracy of prediction results when training time and computational space are limited. Considering the shortcomings of traditional algorithms and reinforcement learning algorithms, scholars have used NIAs, which have high convergence accuracy and strong adaptability.

The NIAs initially proposed and still widely used today include differential evolution (DE) [19] and particle swarm optimization (PSO) [20], and their principles are based on gene exchange and bird foraging, respectively. Scholars have been inspired by human activities, animal behaviors, and mathematical principles to propose more NIAs, including hiking optimization algorithm (HOA) [21], blood-sucking leech optimizer (BSLO) [22], gradient-based optimizer (GBO) [23], and fata morgana algorithm (FATA) [24]. Each original NIA needs further improvement for complex optimization problems. For example, Hu et al. integrated the snow ablation optimizer into PSO framework, resulting in SAOMPSO, which was applied to curve modeling design [25]. Naik et al. proposed the leader slime mold algorithm as a new method used for multilevel thresholding [26]. Tanabe et al. proposed L-SHADE based on a linear function, success history, and adaptive factors [27]. To obtain better solutions using limited computing resources, Aydilek proposed a hybrid algorithm HFPSO, where HF represents the firefly algorithm [28].

In path planning, the goal is to find the shortest safe route for the start–target problem using improved NIAs. Akay et al. proposed an improved differential sine cosine algorithm to improve the efficiency of multirobot path planning [29]. Cao et al. introduced an adaptive DE that balances exploration and exploitation for UAV path planning [30]. Cui et al. developed an improved ABC algorithm for efficient, collision-free paths in multirobot scenarios [31]. Hu et al. proposed a 3D spherical safety corridor design method for fixed wing UAVs based on the super eagle optimization and spherical Said-Ball curve [32]. Huo et al. enhanced ant colony optimization with corner constraints to reduce the blindness of path search [33]. Li et al. proposed a group PSO based on Fermat points and simultaneously optimized the aerial launch positions and segmented paths of composite UAVs [34]. Shankar et al. proposed a hybrid algorithm APFM-PSO for mobile robots in static obstacle environments [35]. Zhao et al. proposed MSS-APSO based on map simplification strategy and adaptive factor and ensured the safe path of autonomous vehicles through safety check strategy and dynamic obstacle avoidance strategy [36]. Scholars study the coverage path planning (CPP) problem of mobile robots alongside the start–target problem [37]. The goal of CPP is to find an optimal path that traverses all points in an area while avoiding obstacles and minimizing path overlap [38]. CPP algorithms can be divided into two categories: offline algorithms and online algorithms based on whether they can grasp environmental information in advance [39]. Online algorithms operate without needing complete map information, utilizing enhanced collision detectors and real-time measurements for target space coverage. Notable examples include random CPP and chaotic CPP, the latter of which generates unpredictable paths to cover unknown areas without localization. First introduced by Nakamura and Sekiguchi in 2001, chaotic CPP has garnered significant attention in civilian and military applications [40]. The chaotic CPP algorithm generates the motion trajectory of robots in the environment through chaotic dynamical systems or maps. A dynamical system is chaotic if it shows sensitive dependence on initial conditions and is topologically transitive.

ETO [41] uses exponential and trigonometric functions as the main body of updating formulas and dynamically adjusts the search range and flexibly switches search strategies through the constraint exploration method and the stage transition method, respectively. Unlike traditional NIAs that rely on animal behaviors or physical laws, ETO solves optimization problems based on mathematical formulas, thereby improving the flexibility and adaptability of the algorithm. Yan et al. used computational fluid dynamics with ETO to address uneven flow and dust deposition in plate dust collectors [42]. Wei et al. developed the ETO Transformer LSTM model to enhance short-term cooling load prediction accuracy in hotels [43]. This article introduces key update formulas from NIAs with strong optimization abilities as strategies into ETO and proposes an improved ETO called IETO for solving the established model. The primary contributions of this paper are as follows:

- Resamples the Bezier curve with a fixed arc length and generates multiple reference points around each resampled point to enhance the safety of paths in spatial structures. Then, a 3D smooth coverage model for UAVs is established, which includes additional path length cost, regional threat cost, terrain collision cost, and smoothness cost.
- This article introduces the alpha evolution strategy (AES), noise and physical attack strategy (NAPAS), and opposition-based cross teaching strategy (OBCTS) into the standard ETO and proposes a new ETO variant called IETO.
- IETO’s potential in complex optimization has been shown through population diversity, ablation analysis, and benchmarks, with its practicality in UAV path planning verified across three flight environments.

This article is structured as follows: Section 2 introduces the method for generating reference points for spherical Bezier curves and establishes a 3D UAV path planning model with four cost functions. Section 3 establishes mathematical models for ETO and IETO. Section 4 analyzes the comprehensive performance of IETO through three experiments and tests its reliability in UAV path planning through simulations in three environments. Finally, Section 5 concludes the article and discusses future work.

## 2. Design of Path Planning Model

In path planning, the model’s design significantly impacts the quality and efficiency of the path. An effective path planning model evaluates paths accurately and guides algorithms to find optimal routes in complex environments. This section introduces our proposed model, which considers factors like path length, regional threats, terrain collision, and smoothness to ensure safe and efficient planning. By optimizing these elements, the model aims to balance various requirements and enhance overall performance.

### 2.1. Overview of Path Planning Model

The cost function *J* is the core of the path planning model, used to quantify the advantages and disadvantages of the path. An excellent cost function should be able to comprehensively reflect the performance of the path in various aspects, thereby guiding the algorithm to make reasonable decisions in complex environments. *J* consists of four subitems, each corresponding to different optimization objectives:

(1) $$J=\sum_{i=1}^{4}k_{i}J_{i},$$

where *J* represents the total cost function. *J*_1_ to *J*_4_ represent the cost of path length, regional threat, terrain collision, and path smoothness, respectively.

### 2.2. Method for Generating Reference Points

In path planning, discretizing a continuous route into multiple reference points is an important step in evaluating and optimizing the path. The distribution and positioning of reference points greatly influence the accuracy of the cost function and path planning. This section will introduce how to generate reference points and their role in the cost function.

Firstly, discretize the route between the start and end points. By using the Bezier curve method to generate the smooth path based on control points, a more accurate path direction can be obtained. The parameterization formula for Bezier curve is as follows [44]:

(2) $$B(t)=\sum_{i=0}^{n}C_{n}^{i}{\left(1-t\right)}^{n-i}t^{i}P_{i}\;,\;t\in [0,1],$$

where $P_{i}$ represents the *i*th control vertex.

Secondly, to ensure a uniform distribution of reference points, we use the equal arc length sampling method to resample the curve with a fixed step size Δs=2R. Assuming the resampling point is $M_{j}$, each $M_{j}$ satisfies that the arc length of the path between adjacent resampling points is approximately 2R. At each resampling point $M_{j}$, a set of reference points distributed around the point is generated based on the tangent vector of the path, and the direction vector $d_{j}$ is obtained after normalizing the tangent vector. At each resampling point $M_{j}$, n=14 reference points are generated, which are located on a sphere with a radius of R centered on $M_{j}$ and evenly distributed. The coordinates of the reference point can be represented as follows:

(3) $$R_{j,k}=M_{j}+R\cdot S_{k}^{\prime }$$

where $S_{k}^{\prime }$ is the rotated unit vector.

To ensure a uniform distribution of reference points, a set of predefined standard unit vectors $S_{k}$ (a total of 14) are selected, and these vectors are rotated according to $d_{j}$ to obtain the rotated unit vector $S_{k}^{\prime }$. $S_{k}^{\prime }$ is defined as follows:

(4) $$S_{k}^{\prime }=\left\{\left(\pm 1,0,0),(0,\pm 1,0),(0,0,\pm 1),(\pm \frac{1}{\sqrt{2}},\pm \frac{1}{\sqrt{2}},0),(\pm \frac{1}{\sqrt{2}},0,\pm \frac{1}{\sqrt{2}}\right)\right\}.$$

These standard unit vectors cover the main directions, including axial and diagonal directions. By rotating matrix $R_{j}$ to align these standard vectors with the tangent vector of the path, we obtain $S_{k}^{\prime }$, ensuring that the reference points are evenly distributed around the path.

Collect all generated reference points $\left\{R_{j,k}\right\}$ to form the reference point set for the entire path:

(5) $$\mathrm{Formation}.\mathrm{refpoint}=\left\{\left.R_{j,k}\right|j=1,2,\ldots ,m;\;\;k=1,2,\ldots ,14\right\}.$$

**Figure 1:**
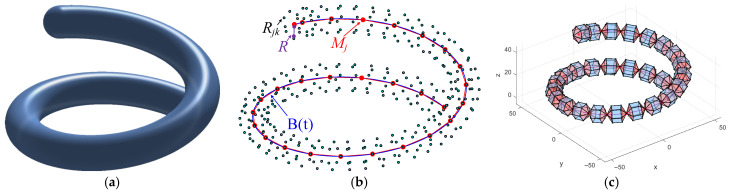
Reference points of the spherical Bezier curve. (**a**) Continuous reference points, (**b**) discrete reference points, (**c**) connected dodecahedron.

This sampling point generation method ensures uniform distribution and comprehensive coverage at various positions in the path, facilitating accurate calculation of the subsequent cost function and not ignoring the importance of certain areas due to uneven reference point density. This method enhances the detection capability of paths for threats and terrain obstacles in different directions by generating multiple reference points around each resampling point. The generation parameters of reference points can be adjusted according to specific application requirements to adapt to the complexity of different environments. The method for generating reference points for the spherical Bezier curve is shown in Figure 1.

### 2.3. Additional Path Length Cost

Path length is a fundamental consideration in path planning, directly affecting the efficiency and energy consumption of navigation. The additional path length cost $J_{1}$ is used to measure the extra length of the path. Firstly, to unify the path length range of the cost function on maps of different sizes, we calculate the length of the standard path $J_{1,s}$:

(6) $$J_{1,s}=\left\|\left[\begin{array}{c} x_{1}-x_{n}\\ y_{1}-y_{n}\\ z_{1}-z_{n} \end{array}\right]\right\|,$$

where $x_{i}$, $y_{i}$, and $z_{i}$ represent the *i*th path point on the *x*-axis, *y*-axis, and *z*-axis, respectively. *n* represents the total number of path points. The standard path is usually the shortest path, with its length serving as a reference benchmark.

Then, calculate the length of the actual path $J_{1,r}$:

(7) $$J_{1,r}=\sum_{i=1}^{n}\left\|\left[\begin{array}{c} x_{i+1}-x_{i}\\ y_{i+1}-y_{i}\\ z_{i+1}-z_{i} \end{array}\right]\right\|,$$

where the actual path length in the formula is obtained by accumulating the Euclidean distances between adjacent path points and fully considering the actual direction of the path and terrain changes.

Finally, the additional path length cost $J_{1}$ is calculated by the following equation and multiplied by a larger constant to ensure its dominant position in optimization:

(8) $$J_{1}=J_{1,\infty }\cdot \left(\frac{J_{1,r}}{J_{1,s}}-1\right),$$

where $J_{1,\infty }$ represents the penalty value. This formula aims to emphasize the importance of path length, avoid planning overly circuitous paths, and ensure the priority of path length in the cost function through the infinite weight. The “⋅” in this paper represents a multiplication sign.

### 2.4. Regional Threat Cost

In practical applications, path planning not only needs to consider the length of the path, but also must avoid potential threat areas to ensure safety. The regional threat cost $J_{2}$ is used to measure the distance between the reference point in the path and the threat area, and the specific calculation steps are as follows:

The security radius of the sphere is *R*, then the total security radius of the *l*th threat area is as follows:

(9) $$R_{safe}^{l}=R+r_{th}^{l},$$

where $r_{th}^{l}$ represents the security radius of the *l*th threat area.

When the distance between the path point of the UAV and the center coordinates of the threat area is greater than $R_{safe}^{l}$, the calculation formula of regional threat cost $J_{2}$ is as follows:

(10) $$J_{2}=\frac{J_{2,\infty }}{N_{th}}\cdot \sum_{p=1}^{P_{num}}\sum_{l=1}^{N_{th}}\frac{\left\|\left[\begin{array}{c} x_{th}^{l}-x_{p}\\ y_{th}^{l}-y_{p} \end{array}\right]\right\|-R_{safe}^{l}}{R_{safe}^{l}},$$

where $J_{2,\infty }$ represents the penalty value. $P_{num}$ represents the number of reference points. $N_{th}$ represents the number of threat areas. $\left(x_{th}^{l},y_{th}^{l}\right)$ represents the center coordinates of the *l*th threat area. $\left(x_{p},y_{p}\right)$ represents the coordinate projection value of the *p*th reference point.

### 2.5. Terrain Collision Cost

The difference in terrain height may lead to collision risks in path planning, especially in uneven or complex terrain environments. The terrain collision cost $J_{3}$ is used to evaluate the relationship between the path reference point and the terrain height, and its calculation process is as follows:

(11) $$J_{3}=\sum_{p=1}^{P_{num}}JZ_{p},$$

(12) $$JZ_{p}=\left\{\begin{array}{cc} 0 & Z_{p}-Zmap_{p}\geq Z_{safe}\\ J_{3,\infty }\cdot \left(1-\frac{Z_{p}-Zmap_{p}}{Z_{safe}}\right) & 0<Z_{p}-Zmap_{p}<Z_{safe}\\ J_{3,\infty }\cdot e^{1+\left(Zmap_{p}-Z_{p}\right)} & \;Z_{p}-Zmap_{p}\leq 0 \end{array}\right.,$$

where $J_{3,\infty }$ represents the penalty value. $Z_{safe}$ represents the safe height. $Zmap_{p}$ represents the terrain height corresponding to the *p*th reference point.

### 2.6. Smooth Cost

The smoothness of a path impacts navigation stability. A smoother path minimizes sharp turns and vibrations, enhancing navigation performance. The smoothness cost $J_{4}$ is calculated using the path’s curvature and torsion, based on its first, second, and third derivatives.

Firstly, calculate the derivative of the position vector $f\left(s\right)$ of each point on the path with respect to the parameter *s*:

(13) $$f^{\prime }(s)=\frac{df}{ds},\;f^{\prime \prime }(s)=\frac{d^{2}f}{ds^{2}},\;f^{\prime \prime \prime }(s)=\frac{d^{3}f}{ds^{3}},$$

where $f\left(s\right)$ represents the position vector of the path under parameter *s*. *s* represents the path parameter, which is proportional to the arc length of the path and is used to parameterize the path. By parameterizing the path, its derivative can be calculated more conveniently.

The calculation formulas for curvature κ and torsion τ are as follows:

(14) $$\kappa =\frac{\left\|f^{\prime }\left(s\right)\times f^{\prime \prime }\left(s\right)\right\|}{{\left\|f^{\prime }\left(s\right)\right\|}^{3}},$$

(15) $$\tau =\frac{\left\|f^{\prime \prime \prime }\left(s\right)\cdot \left(f^{\prime }\left(s\right)\times f^{\prime \prime }\left(s\right)\right)\right\|}{{\left\|f^{\prime }\left(s\right)\times f^{\prime \prime }\left(s\right)\right\|}^{2}},$$

where κ describes the degree of curvature of the path at a certain point, while τ describes the degree of distortion of the path. In Equations (14) and (15), “⋅” and “×” represent dot multiplication and cross multiplication, respectively.

The smooth cost $J_{4}$ is enhanced in optimization by taking the maximum absolute values of κ and τ and multiplying them by $J_{4,\infty }$. The final formula for calculating the smooth cost $J_{4}$ is as follows:

(16) $$J_{4}=J_{4,\infty }\cdot \left(\max\left|\kappa \right|+\max\left|\tau \right|\right),$$

where $\max\left|\kappa \right|$ represents the maximum absolute curvature value of all points on the path, reflecting the most curved part of the path. $\max\left|\tau \right|$ represents the maximum absolute value of the torsion of all points on the path, reflecting the most twisted part of the path.

This design ensures that the path has good smoothness on κ and τ, avoiding sharp turns and distortions, thereby improving the comfort and stability of navigation. Meanwhile, by introducing the weight $J_{4,\infty }$, the importance of smoothness is further emphasized, making the path planning algorithm pay more attention to the geometric shape of the path during optimization.

## 3. Standard and Improved ETO

This section introduces the standard ETO in Section 3.1 and presents a new variant, IETO, in Section 3.2.

### 3.1. Standard ETO

#### 3.1.1. Constraint Exploration Method

In optimization, algorithm performance affects the search space size. A large search space can slow convergence and waste resources, while a small one may miss the global optimal solution. ETO tackles this by dynamically adjusting search space limits using a constraint exploration method, ensuring efficient searches without missing optimal solutions.

The core of the constraint exploration method lies in dynamically calculating a new iteration constraint and updating the upper and lower limits of the search space. The calculation of $CE_{t}$ is as follows:

(17) $$CE_{t}=\mathrm{floor}\left[2\cdot t\cdot \left(T-a\cdot c\right)\right]+c,$$

(18) $$c=\mathrm{floor}\left[1+T/b\right],$$

where *t* is the current iteration number. *T* is the maximum number of iterations. *a* and *b* are adjustment coefficients and are set to constants of 4.6 and 1.55, respectively. $CE_{t}$ is the new iteration constraint. ETO dynamically calculates $CE_{t}$ to limit the expansion or contraction of the search space, thereby reducing the consumption of computing resources.

After determining the new $CE_{t}$, the upper and lower limits are updated using the following formulas:

(19) $$Up_{t}=r_{1}\cdot \left(1-\frac{t}{T}\right)\cdot \left|r_{2}\cdot X_{best}^{t}-X_{s}^{t}\right|+X_{best}^{t},$$

(20) $$Low_{t}=-r_{1}\cdot \left(1-\frac{t}{T}\right)\cdot \left|r_{2}\cdot X_{best}-X_{s}\right|+X_{best},$$

where $Up_{t}$ and $Low_{t}$ are the upper and lower limits of the search space, respectively. $r_{j}$ is a random number within the range of (0,1), j=1,2,…,26. $X_{best}^{t}$ and $X_{s}^{t}$ represent the optimal and suboptimal solutions of the *t*th generation, respectively.

#### 3.1.2. Initialization Stage

NIAs typically assign values to the initial population using the following formula:

(21) $$X_{i}=Lb+(Ub-Lb),$$

where ***Lb*** and ***Ub*** are the lower and upper bounds. $X_{i}$ is the *i*th individual in the initial population, i=1,2,…,N. *N* is the population size.

#### 3.1.3. Exploration Stage

The exploration stage focuses on searching for new potential areas while approaching the optimal solution. It consists of two sub-stages, each with a unique strategy to enable global search and avoid local optimal.

Stage 1: In the early iterations, individuals explore a larger search space to find potential solutions. The goal is to cover a wider area and increase the chances of locating the global optimal solution. The simulation formula is as follows:

(22) $$X_{i}^{t+1}=\left\{\begin{array}{cc} X_{best}^{t}+\alpha_{1}\cdot r_{3}\cdot \left|X_{best}^{t}-X_{i}^{t}\right|, & \mathrm{if}\;r_{4}\leq 0.5\\ X_{best}^{t}-\alpha_{1}\cdot r_{3}\cdot \left|X_{best}^{t}-X_{i}^{t}\right|, & \mathrm{else} \end{array}\right.,$$

(23) $$\alpha_{1}=3\cdot r_{5}\cdot \left(\frac{t}{T}-0.85\right)\cdot \exp\left(-2\right),$$

where $\alpha_{1}$ is the first weight coefficient used to control the distance between individual movements.

ETO optimizes search efficiency by combining $\alpha_{1}$ and the exponential function, reducing the movement distance of individuals over iterations. This approach allows for broad coverage of the search space initially, then focuses on the area near the optimal solution, enhancing convergence speed and accuracy.

Stage 2: To further explore new areas, the simulation formula in this stage is as follows:

(24) $$X_{i}^{t+1}=\left\{\begin{array}{cc} X_{best}^{t}+\alpha_{2}\cdot r_{6}\cdot \left|X_{best}^{t}-X_{i}^{t}\right|, & \mathrm{if}\;r_{7}\leq 0.5\\ X_{best}^{t}-\alpha_{2}\cdot r_{6}\cdot \left|X_{best}^{t}-X_{i}^{t}\right|, & \mathrm{else} \end{array}\right.,$$

(25) $$\alpha_{2}=r_{8}\cdot \exp\left\{\tanh\left[1.5\cdot \left(-\frac{t}{T}-r_{9}\right)\right]\right\},$$

where $\alpha_{2}$ is the second weight coefficient. $\alpha_{2}$ fluctuates with the increase of *t* to ensure that ETO performs a more detailed search when approaching the optimal solution.

#### 3.1.4. Exploitation Stage

ETO conducts a targeted search near the optimal solution during the exploitation stage, which consists of two sub-stages to improve solution quality.

**Figure 2:**
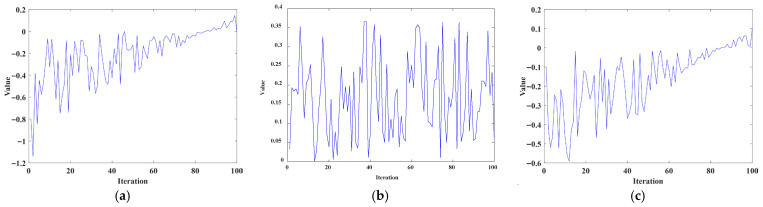
The variation of three weight coefficients during iteration. (**a**) The change of $\alpha_{1}$, (**b**) the change of $\alpha_{2}$, and (**c**) the change of $\alpha_{3}$.

Stage 1: ETO enhances solution accuracy by concentrating on the local search around the current optimal solution. The simulation formula is as follows:

(26) $$X_{i}^{t+1}=\left\{\begin{array}{cc} X_{best}^{t}+\alpha_{3}\cdot r_{10}\cdot r_{11}\cdot \left|X_{best}^{t}-X_{i}^{t}\right|, & \mathrm{if}\;r_{12}\leq 0.5\\ X_{best}^{t}-\alpha_{3}\cdot r_{13}\cdot r_{11}\cdot \left|X_{best}^{t}-X_{i}^{t}\right|, & \mathrm{else} \end{array}\right.,$$

(27) $$\alpha_{3}=3\cdot r_{14}\cdot \left(\frac{t}{T}-0.85\right)\cdot \exp\left(-2.3\right),$$

where $\alpha_{3}$ is the third weight coefficient. As *t* increases, $\alpha_{3}$ gradually decreases to ensure that ETO performs a more detailed search when approaching the optimal solution. The numerical changes of $\alpha_{1}$, $\alpha_{2}$, and $\alpha_{3}$ during iteration are shown in Figure 2.

Stage 2: ETO focuses on a deep search near the optimal solution to enhance stability:

(28) $$X_{i}^{t+1}=X_{i}^{t}+h\cdot r_{15}\cdot \alpha_{2}\cdot \left|X_{best}^{t}-X_{i}^{t}\right|,$$

(29) $$h=\exp\left[\tan\left(1\right)\right],$$

where *h* is the coefficient that controls the movement interval. By combining with exponential and tangent functions, the search process of ETO has sufficient flexibility and depth.

#### 3.1.5. Stage Transition Method

An efficient transition mechanism is needed during the exploration and exploitation stages to ensure that ETO can extensively search and deeply optimize. Firstly, set the constant *ST* = floor [1.2 + *T*/2.25] to switch between the exploration and exploitation stages. Then, ETO achieves smooth switching between the first and second stages using the following adaptive factor:

(30) $$AF=0.01\cdot r_{16}\cdot \sqrt{\frac{t}{T}}\cdot \tan\left(-1\right),$$

where *AF* is the adaptive factor for controlling the stage transition. When *AF* < 1, ETO is in the first stage, otherwise it is in the second stage. *AF* enables ETO to flexibly switch search strategies during the iteration process, effectively avoiding ETO falling into the local optimal solution.

### 3.2. The New ETO Variant

#### 3.2.1. Alpha Evolution Strategy

In NIAs, excellent search operators are crucial for guiding algorithms to discover higher quality solutions. The AES can achieve efficient searching through a single *α* operator, which integrates multiple steps into a single arithmetic unit by synchronizing extraction and evolution information [45], as follows:

(31) $$X_{i}^{t+1}=X_{best}^{t}+\alpha \cdot RSS_{i}^{t}+\theta \cdot \left(EI_{i}^{t}+X_{i}^{t}-X_{best}^{t}-OI_{i}^{t}\right),$$

(32) $$\alpha =\exp\left[\ln\left(\frac{T-t}{T}\right)-{\left(\frac{4\cdot t}{T}\right)}^{2}\right],$$

(33) $$RSS_{i}^{t}=\left(Up_{t}-Low_{t}\right)\cdot \left(2\cdot R_{1}\cdot R_{2}-R_{2}\right)\cdot S,$$

(34) $$\theta =l\cdot R_{3}+(1-l)\cdot 2\cdot r_{17},$$

where *α* is the attenuation factor. $RSS_{i}^{t}$ represents the *i*th row in the random step matrix. θ is used to adjust the adaptive step size, where *l* is randomly taken as 0 or 1. $EI_{i}^{t}$ represents a random elite individual with better fitness than $X_{i}^{t}$, while $OI_{i}^{t}$ represents a random ordinary individual with worse fitness than $X_{i}^{t}$. ***R***_1_, ***R***_2_, and ***R***_3_ are three matrices composed of random numbers within (0, 1), with sizes of *N* × *D*, *N* × *D*, and 1 × *D*. ***S*** only contains 0 and 1, with the size of *N* × *D*.

Equation (33) can be divided into three parts. $X_{best}$ in the first part is the benchmark for individual movement in the AES. The multiple random vectors included in the second part provide global exploration ability for ETO. The sum of the two sets of differential vectors in the third part provides local exploitation ability for ETO. The AES improves the optimization ability of ETO by combining three parts as a single arithmetic unit.

#### 3.2.2. Noise and Physical Attack Strategy

In this section, the noise strategy and physical attack strategy are introduced into the exploration and exploitation stages of ETO, respectively, and NAPAS is proposed [46]. The noise strategy simulates porcupines creating noise to deter predators, and this strategy can help ETO find potential areas that may contain global optimal solutions. The simulation formula for the noise strategy is as follows:

(35) $$X_{i}^{t+1}=\left(1-U_{1}\right)\cdot X_{i}^{t}+U_{1}\cdot \left(\left(\frac{X_{i}^{t}+X_{l_{1}}^{t}}{2}\right)+r_{18}\cdot \left(X_{l_{1}}^{t}-X_{l_{2}}^{t}\right)\right),$$

where *l*_1_ and *l*_2_ are two random integers taken from [1, *N*], and $l_{1}\neq l_{2}\neq i$. ***U***_1_ is a vector containing 0 and 1. When the element in ***U***_1_ is 1, it represents that the predator remains stationary in its original position. When the element in ***U***_1_ is 0, it represents that the predator decides to approach or move away from the prey based on the dimensional difference between two random individuals.

The physical attack strategy simulates porcupines using their fur to fight against predators, and this strategy can help ETO further explore the potential areas found before. The simulation formula for physical attack strategy is as follows:

(36) $$X_{i}^{t+1}=X_{best}^{t}+\left(0.2\cdot \left(1-r_{19}\right)+r_{19}\right)\cdot \left(U_{2}\cdot X_{best}^{t}-X_{i}^{t}\right)-r_{20}\cdot U_{2}\cdot F_{i}^{t}\cdot 2\cdot r_{21}\cdot {\left(1-t/T\right)}^{t/T},$$

(37) $$F_{i}^{t}=r_{22}\cdot \left(X_{l_{3}}^{t}-X_{i}^{t}\right),$$

where $F_{i}^{t}$ represents the interaction force corresponding to the *i*th individual in the *t*th generation. ***U***_2_ is a vector containing 0 and −1. *l*_3_ is a random integer taken from [1, *N*], and $l_{3}\neq i$.

#### 3.2.3. Opposition-Based Cross-Teaching Strategy (OBCTS)

The teaching strategy that incorporates random individuals is introduced into ETO to enrich its exploration mechanism [47], and the OBCTS further uses three randomly selected individuals to perturb the values of each dimension. ETO enhances behavioral diversity through a cross-teaching strategy and a position update formula using random individuals. The cross-teaching process is as follows:

(38) $$X_{i}^{t+1}=\left\{\begin{array}{cc} X_{l_{4}}^{t}+r_{23}\cdot [X_{l_{5}}^{t}-X_{l_{6}}^{t}] & \mathrm{if}\;r_{24}\leq CR\\ X_{i}^{t}+r_{25}\cdot [X_{best}^{t}-TF\cdot X_{mean}^{t}] & \mathrm{else} \end{array}\right.,$$

where *CR* is the crossover rate and is determined to be a constant of 0.7 in Section 4.2. $X_{mean}^{t}$ represents the average position of the population. *TF* is a teaching factor, randomly selected as 1 or 2. *l*_4_, *l*_5_, and *l*_6_ are three random integers taken from [1, *N*], and $l_{4}\neq l_{5}\neq l_{6}\neq i$.

Then, this section introduces the opposition-based learning strategy (OBLS) into ETO [48]. The OBLS can generate the opposite individuals corresponding to each individual, and scholars have found that introducing random vectors into OBLS can fine-tune the position of opposite individuals [49]. This section introduces the coefficient matrix with richer random vectors from reference [50] into the OBLS and calculates the opposite population ***OX*** as follows:

(39) $$OX_{i}^{t}=Low_{t}+Up_{t}-CM_{k}\cdot X_{i}^{t+1},$$

(40) $$CM_{s}=\left\{\begin{array}{cc} R_{4}, & s=1\\ -t/T+R_{5}, & s=2\\ \left(-1-t/T\right)\cdot H_{1}, & s=3\\ H_{2}\cdot {\left(H_{3}\right)}^{2}\cdot \cos\left(2\cdot r_{26}\cdot H_{2}\right), & s=4 \end{array}\right.,$$

where $OX_{i}^{t}$ represents the opposite individual of the *i*th individual in the *t*th generation. *k* is an index used to randomly select a vector in the coefficient matrix ***CM***, and $k\in \left[1,2,3,4\right]$. ***R***_4_ and ***R***_5_ are two vectors composed of random numbers within (0, 1), both with the size of 1 × *D*. ***H***_1_, ***H***_2_, and ***H***_3_ are three random vectors under the standard normal distribution.

#### 3.2.4. Computational Complexity Analysis

The computational complexity of IETO is affected by *N*, *D*, and *T*. The parts related to this value in IETO include the update formulas in the standard ETO *O*(ETO), the alpha evolution strategy *O*(AES), the noise and physical attack strategy *O*(NAPAS), and the opposition-based cross teaching strategy *O*(OBCTS). In addition to the key individual update formulas, the computational complexity also needs to consider the updating of adaptive parameters and random arrays, and the calculation results are as follows:

(41) $$\begin{array}{ll} O(\mathrm{IETO}) & =O(\mathrm{ETO})+O(\mathrm{AES})+O(\mathrm{NAPAS})+O(\mathrm{OBCTS})\\ & =O(TND+3T)+O(TND+2TD+T)+O(TND+2TD+2T)+O(2TND+5TD)\\ & =O(6T+9TD+5TND) \end{array}.$$

**Figure 3:**
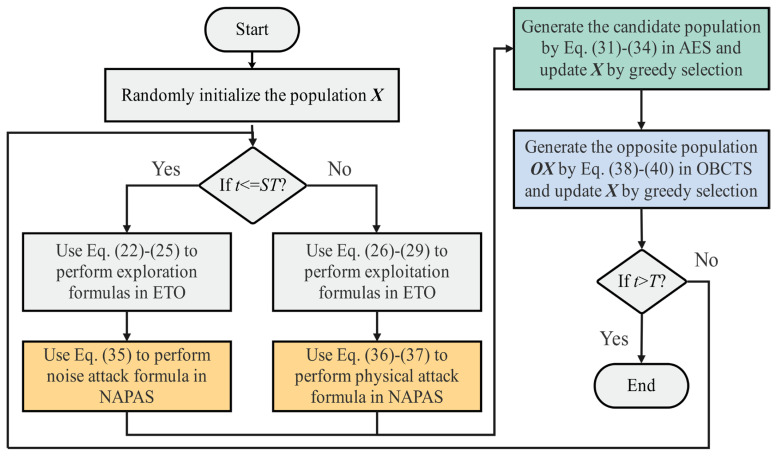
The flowchart of IETO.

The flowchart of IETO is shown in Figure 3, and its pseudo-code is shown in Algorithm 1.
**Algorithm** **1.** The pseudo-code of IETO.**Input:** *N*, *D* and *T***Output:** The optimal individual ***xbest*** and its corresponding fitness value *fbest*1: Initialize the population ***X*** by Equation (21)2: **For** *t* = 1:*T*3:      **If** *t* <= *ST*4:         Use Equations (22)–(25) to perform exploration formulas in ETO5:      Use Equation (35) to perform noise attack formula in NAPAS6:     **Else**7:      Use Equations (26)–(29) to perform exploitation formulas in ETO8:      Use Equations (36) and (37) to perform physical attack formula in NAPAS9:     **End If**10:    Generate the candidate population by Equations (31)–(34) in AES and update ***X***11:    Generate the opposite population ***OX*** by Equations (38)–(40) in OBCTS and update ***X***12:    Check if each solution is within the boundary and its fitness value is calculated13:    Update ***xbest*** and *fbest*14: **End For**

## 4. Experimental Results of IETO

Section 4.1 determines the environment, parameters, and comparison algorithms required for experiments. Section 4.2 analyzes population diversity in IETO and ETO, while Section 4.3 conducts an ablation analysis on ETO variants. Section 4.4 highlights the superior performance of IETO, and Section 4.5 evaluates its effectiveness across three simulated topographies.

### 4.1. Experimental Setup

We selected the 50 dimensional CEC2020 test set as the experimental environment. Besides the standard ETO, the comparison algorithms include recently proposed new algorithms such as HOA, BSLO, GBO, and FATA, along with other efficient, improved algorithms including SAOMPSO, LSMA, L-SHADE, and HFPSO. Table 1 documents the constant settings for the comparison algorithms. This section sets parameters *N*, *T*, and *RN* to 100, 1000, and 30, respectively, where *RN* represents the number of runs for each algorithm.

### 4.2. Population Diversity Analysis

Population diversity can quantify the performance differences between algorithms [51]. The algorithm with a stronger exploration ability will correspond to a slower decreasing curve, and the population diversity calculation is as follows:

(42) $$Div^{t}=\frac{1}{N\times SZ}\sum_{i=1}^{N}\sqrt{\sum_{j=1}^{D}{\left(X_{i,j}^{t}-X_{mean,j}^{t}\right)}^{2}}$$

where *SZ* represents the maximum exploration range of algorithms.

**Figure 4:**
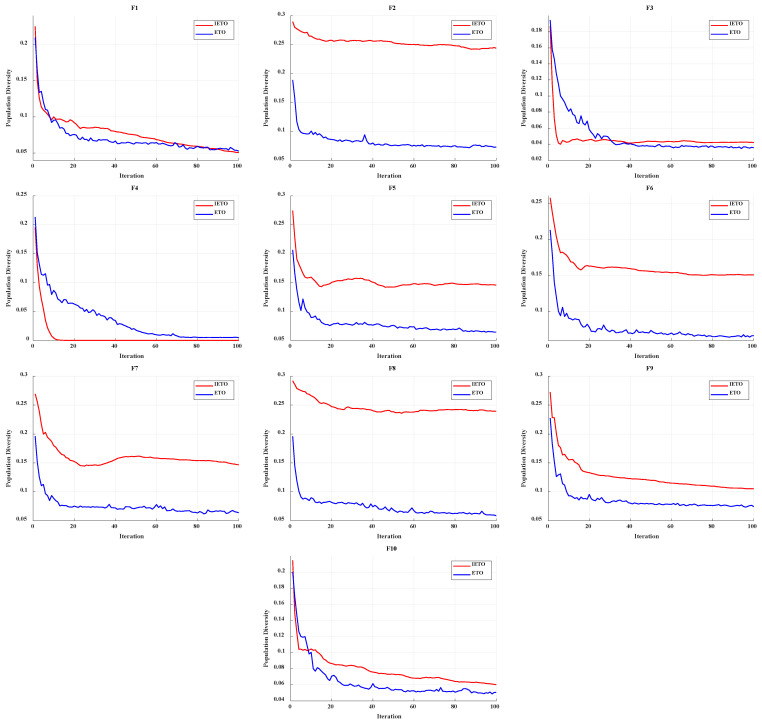
Average diversity curves for IETO and ETO.

Figure 4 shows the average diversity curves of ETO and IETO, with only the first 100 iterations plotted for comparison. From Figure 4, the red curve representing IETO consistently outperforms the blue curve for ETO, demonstrating the effectiveness of the strategies in IETO. The red curve exhibits no rapid decline for the majority of functions, which proves that IETO can maintain higher exploration ability for a long time.

### 4.3. Ablation Analysis

Table 2 shows various ETO variants, where 1 and 0, respectively, represent introducing the current strategy into ETO or not. Table 3 records the ablation experimental results, with optimal values highlighted in bold black.

From Table 3, all ETO variants outperform the basic ETO. With the increase of introduced strategies, ETO variants achieve more optimal values. OETO, AOETO, and NPETO demonstrate significant advantages over other ETO variants, indicating that the OBCTS provides the greatest enhancement to the optimization ability of ETO. IETO achieves optimal values on 80% of the functions, validating the effectiveness of incorporating the three strategies within IETO.

### 4.4. Benchmark Experiment

**Figure 5:**
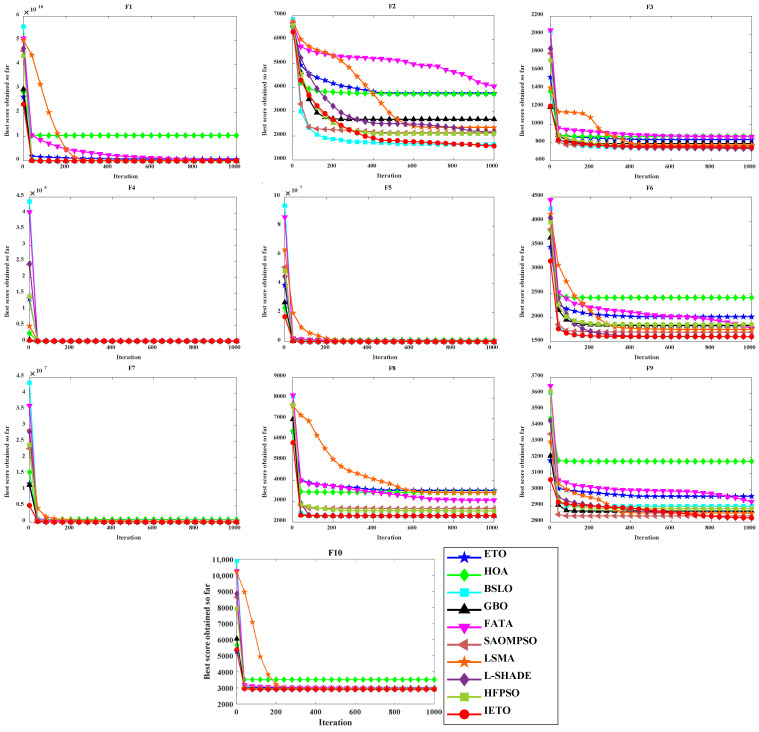
Average convergence curves.

**Figure 6:**
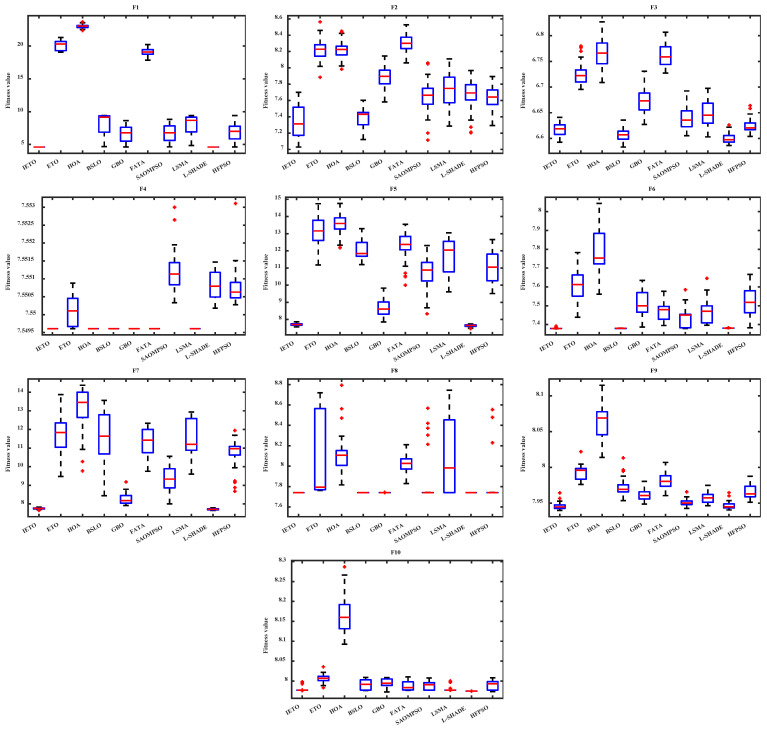
Box plots.

Table 4 records the results of the benchmark experiment, with optimal values highlighted in bold. OV, MV, and STD, respectively, record the optimal value, mean value, and standard deviation of the fitness array. The *p*-value is based on the degree of difference between two arrays and used to determine the superiority or inferiority of IETO and the comparison object, with symbols “+”, “-”, and “=“ representing superiority, inferiority, and no difference, respectively [52]. MR is the mean rank of each algorithm based on the Friedman test [53]. Figure 5 and Figure 6 display the average convergence curves and box plots, respectively.

For the unimodal function F1, IETO achieves optimal values on OV, MV, STD, and MR, with all WRST results being “+”. Both the OV and MV of IETO are 1.0000 × 10^2^, matching the global optimal value of F1 itself. This demonstrates that IETO can provide higher-precision solutions for optimization problems with simple function structures. In F2–F4, BSLO and L-SHADE pose significant threats to IETO, and the WRST results of IETO against these two algorithms in F3 are all “-”. IETO demonstrates clear advantages in performance indexes in F2 and F4 but consistently ranks third in F3. In F5–F7, IETO only achieves suboptimal MV and STD values compared to BSLO in F6. The WRST results, which consist solely of “+” symbols, demonstrate that IETO exhibits superior overall performance in optimization problems with higher complexity.

As composition functions, F8–F10 construct new functions with complex properties by nested use of multiple functions. IETO fails to achieve optimal values for all performance metrics in F8–F10. The WRST results indicate that IETO performs worse than L-SHADE in F8 and F10, corresponding to its two suboptimal MR. From the performance of IETO in Figure 5, the convergence curve of IETO descends to the bottom faster than comparison algorithms, and it does not rapidly stabilize in the later iterations. In addition, the box of IETO in Figure 6 is always the narrowest and lowest, indicating that it can stably provide higher quality solutions.

### 4.5. Simulation Experiment

In this experiment, we employ three simulated topographic maps to test algorithms. These maps encompass a range of requirements from basic terrain testing to complex environment simulations, utilizing a flexible and controllable generation method to adapt to diverse research and application contexts. All test environments are assigned specific start points, and each path contains four control vertices.

Map1 displays a simple terrain featuring a prominent mountain peak, which is presented on a 512 × 512 grid and covers an area of 1000 units. The single peak with a broad base forms a distinct elevation feature, ideal for studying a single primary terrain element. Map2 displays a highly complex and diverse terrain composed of fifty independent peaks, which is also presented on a 512 × 512 grid and covers an area of 1000 units. Each peak stands 300 to 400 m tall with a base size of 20 to 40 units. Peaks are densely and orderly spaced at least 150 units apart, simulating a realistic mountainous region. Map2 is suitable for advanced simulations and research such as agent navigation, terrain analysis, or ecological study. Map3 generates complex and detailed terrain through random Fourier transforms, which covers a 200 × 200 grid and a coordinate range of 1000 units. There are ten preset threat points with specific locations and intensities in the map to simulate high-risk zones. Map3 is suitable for setting fixed points of interest in complex terrains, such as tactical simulation or defense strategy evaluation.

This section systematically evaluates the stability and robustness of IETO under varying safety radius (Sr) and safety height (Sh). Three maps are selected for experimentation, with Sr set to 5, 10, 15, and 20 m, and Sh set to 5, 10, 15, and 20 m, yielding a total of 16 parameter combinations. To ensure statistical reliability, each combination is run 30 times. All experiments are conducted on the MATLAB 2023b simulation platform. Table 5 shows the statistical results of the robustness experiments of IETO on three maps, where the values before and after the “+” sign represent the mean and standard deviation of IETO, respectively. In three cases, the average fitness value usually increases with the increase of Sr and Sh, which is consistent with the design of the fitness evaluation model. Regarding the standard deviation, the combination of Sr = 10 and Sh = 10 yields a smaller standard deviation on Map1 and Map2, which is also the reason why this combination is chosen as the constant in this article. On Map3, although Sh = 5 shows greater stability, considering the observed trends in other combinations and maps, we ultimately select the combination of Sr = 10 and Sh = 10. The results of IETO in Table 5 demonstrate its strong robustness under dynamic changing conditions.

Table 6 presents the numerical results of all algorithms across three simulated terrains, with optimal values highlighted in bold. From Table 6, IETO obtains the optimal feasible paths in Map1 and Map3, and the corresponding fitness values are 16.7% and 8.1% lower than the suboptimal algorithm, respectively. IETO obtains the suboptimal feasible path in Map2, and the corresponding fitness value is 11.0% higher than that of L-SHADE.

**Figure 7:**
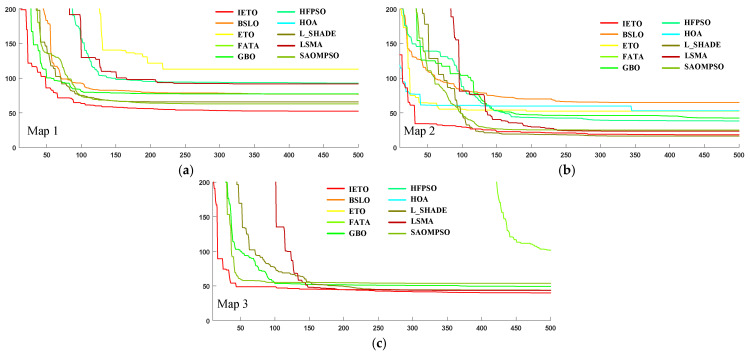
Convergence curve chart. (**a**) Map1, (**b**) Map2, (**c**) Map3.

Figure 7 illustrates the fitness value trends of all algorithms across three simulated terrains. In Map1 and Map3, IETO demonstrates optimal fitness values throughout the optimization stage, outperforming the other nine comparison algorithms. In Map2, the optimization ability of IETO is slightly inferior to L-SHADE, ranking second among all algorithms. This indicates the ability of IETO to find near-optimal paths across diverse terrain types. Furthermore, IETO finds near-optimal solutions within 50 iterations on both Map2 and Map3, demonstrating its strong ability to rapidly identify optimal solutions in complex terrains.

**Figure 8:**
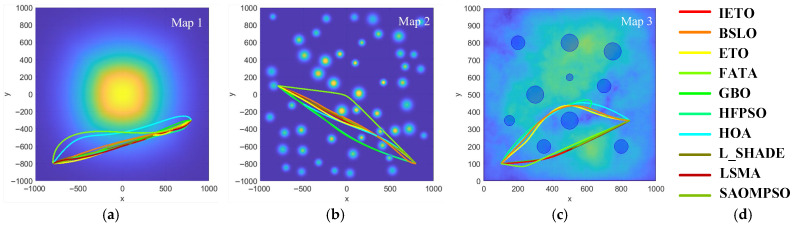
Top view. (**a**) Map1, (**b**) Map2, (**c**) Map3, (**d**) legend.

**Figure 9:**
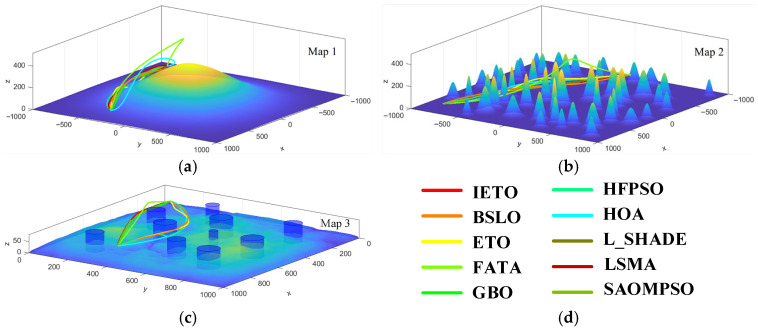
Side view. (**a**) Map1, (**b**) Map2, (**c**) Map3, (**d**) legend.

Figure 8 and Figure 9 display the top view and the side view of optimal paths generated by all algorithms across three simulated terrains, respectively. In Map1, FATA and HOA exhibit noticeable detouring behavior, indicating these algorithms struggle to identify suitable feasible paths even in simple planning scenarios. In Map2, FATA exhibits an abnormal height protrusion, while GBO and HFPSO also bypass longer distances in the horizontal direction, indicating the inadequate ability of these algorithms for path planning in complex scenarios. In Map3, HFPSO, HOA, BLSO, and ETO bypass two threat zones, indicating that these algorithms are prone to local optimal in this scenario. In Map3, except for FATA, other algorithms have little difference and can all find near-optimal feasible paths. From three simulated experiments, IETO consistently provides smoother and shorter safe flight paths for UAVs.

## 5. Conclusions and Future Works

This article presents a smooth path planning method named IETO for UAVs, which solves the problem of the insufficient adaptability of traditional path planning methods by combining spherical Bezier curve and multiple strategies. The introduction of the fixed arc length resampling strategy significantly improves the safety and smoothness of the path, and the comprehensive optimization model considers additional path length, regional threat, terrain collision, and smoothness. The results show that IETO performs well in both benchmark and simulation experiments, and its generated paths are close enough to the ideal length and significantly improve smoothness, which proves its robustness and superiority in complex environments.

This study has yielded positive results; however, it is important to acknowledge certain limitations. Our research and experiments do have the following limitations: Currently, validation has only been conducted using three artificial terrain maps in a simulation environment, without flight testing on an actual UAV platform. Therefore, we cannot yet quantify the impact of aerodynamic interference, turn speed and acceleration limits, control system response delays, and physical constraints of servos and engines on the tracking of smooth Bézier curves. Additionally, we have not thoroughly analyzed the detrimental effects of actuator noise, GPS positioning errors, and inertial navigation system drift on trajectory accuracy. To address these issues, our future work will focus on three key areas: First, conducting phased flight tests in both indoor controlled environments and outdoor open environments to evaluate and isolate the impact of various physical factors. Second, introduce multi-sensor fusion (GPS + IMU + vision/lidar) and robust control strategies to enhance tracking accuracy through closed-loop correction and drift compensation. Third, integrate incremental SLAM with online environment update modules to enable real-time perception and path re-planning for dynamic obstacles and information-deficient scenarios, thereby strengthening system stability and adaptability in complex environments. Additionally, to meet real-world mission demands for real-time performance and multi-UAV coordination, we must evaluate algorithm runtime latency on typical UAV onboard computing platforms (e.g., embedded ARM, flight controllers, or processors with GPU/DSP) and reduce computational overhead through C/C++ optimization, parallel computing, or hardware acceleration to ensure online replanning capability and extend single-UAV path planning to multi-UAV cooperative scenarios by designing centralized and distributed collaborative planning and collision avoidance mechanisms. These address communication latency, task allocation, and trajectory conflicts to achieve joint multi-UAV mission planning and safe cooperative flight. Through these enhancements, the proposed method demonstrates greater feasibility and practical value in real-world dynamic, multi-UAV cooperative environments.

## Figures and Tables

**Table 1:** Constant settings.

Algorithm	Parameter	Value
HOA	Maximum tilt angle *θ*_max_	50
BSLO	Proportional parameter *m*	0.8
	Minimum disturbance control coefficient *dc*_min_	0.001
GBO	Local escape probability *PR*	0.5
FATA	Reflectance *r*	0.2
SAOMPSO	Subgroup proportion *P*	0.5
LSMA	Elimination and diffusion rate *z*	0.03
L-SHADE	Subpopulation size *H*	6
HFPSO	Absorption efficiency *γ*	1
	Attraction efficiency *β*	2
	Randomization factor *φ*	0.2
	Acceleration factor *C*	1.49445

**Table 2:** Various ETO variants.

Strategy	ETO	AETO	NETO	OETO	ANETO	AOETO	NOETO	IETO
AES	0	1	0	0	1	1	0	1
NAPAS	0	0	1	0	1	0	1	1
OBCTS	0	0	0	1	0	1	1	1

**Table 3:** Ablation experiment.

F	ETO	AETO	NETO	OETO	ANETO	AOETO	NOETO	IETO
1	8.7583 × 10^8^	2.3889 × 10^2^	5.4759 × 10^3^	9.9020 × 10^2^	3.8418 × 10^2^	1.0000 × 10^2^	2.6091 × 10^2^	1.0000 × 10^2^
2	3.5973 × 10^3^	1.9564 × 10^3^	1.7776 × 10^3^	1.8165 × 10^3^	1.7057 × 10^3^	1.5277 × 10^3^	1.8037 × 10^3^	1.5834 × 10^3^
3	8.4125 × 10^2^	7.7826 × 10^2^	7.5705 × 10^2^	7.7695 × 10^2^	7.4899 × 10^2^	7.5589 × 10^2^	7.5277 × 10^2^	7.4823 × 10^2^
4	1.9007 × 10^3^	1.9035 × 10^3^	1.9031 × 10^3^	1.9000 × 10^3^	1.9028 × 10^3^	1.9000 × 10^3^	1.9000 × 10^3^	1.9000 × 10^3^
5	5.2893 × 10^5^	2.6579 × 10^3^	2.6566 × 10^5^	5.9203 × 10^3^	2.6073 × 10^3^	2.2179 × 10^3^	4.3385 × 10^3^	2.1997 × 10^3^
6	2.0033 × 10^3^	1.7152 × 10^3^	1.6745 × 10^3^	1.6587 × 10^3^	1.6282 × 10^3^	1.6196 × 10^3^	1.6104 × 10^3^	1.6031 × 10^3^
7	2.0199 × 10^5^	2.6134 × 10^3^	1.2969 × 10^5^	2.7490 × 10^3^	2.5476 × 10^3^	2.3789 × 10^3^	2.6998 × 10^3^	2.3529 × 10^3^
8	3.1994 × 10^3^	2.7970 × 10^3^	2.7346 × 10^3^	2.3000 × 10^3^	2.6510 × 10^3^	2.3000 × 10^3^	2.3000 × 10^3^	2.3000 × 10^3^
9	2.9464 × 10^3^	2.8309 × 10^3^	2.8909 × 10^3^	2.8534 × 10^3^	2.8313 × 10^3^	2.8262 × 10^3^	2.8752 × 10^3^	2.8199 × 10^3^
10	2.9957 × 10^3^	2.9178 × 10^3^	2.9448 × 10^3^	2.9170 × 10^3^	2.9279 × 10^3^	2.9178 × 10^3^	2.9346 × 10^3^	2.9189 × 10^3^

**Table 4:** Statistical results of benchmark experiment.

F	Index	IETO	ETO	HOA	BSLO	GBO	FATA	SAOMPSO	LSMA	L-SHADE	HFPSO
1	OV	1.000 × 10^2^	1.926 × 10^8^	5.230 × 10^9^	1.065 × 10^2^	1.010 × 10^2^	5.613 × 10^7^	1.029 × 10^2^	1.256 × 10^2^	1.000 × 10^2^	1.007 × 10^2^
	MV	1.000 × 10^2^	6.974 × 10^8^	1.050 × 10^10^	6.885 × 10^3^	1.318 × 10^3^	2.312 × 10^8^	1.853 × 10^3^	5.568 × 10^3^	1.000 × 10^2^	2.304 × 10^3^
	STD	1.266 × 10^−14^	4.561 × 10^8^	3.112 × 10^9^	5.009 × 10^3^	1.324 × 10^3^	1.330 × 10^8^	2.198 × 10^3^	4.228 × 10^3^	3.100 × 10^−5^	3.057 × 10^3^
	*p*-value	\	1.510 × 10^−11^ +	1.510 × 10^−11^ +	1.510 × 10^−11^ +	1.510 × 10^−11^ +	1.510 × 10^−11^ +	1.510 × 10^−11^ +	1.510 × 10^−11^ +	1.510 × 10^−11^ +	1.510 × 10^−11^ +
	MR	1.00	8.87	10.00	6.07	4.40	8.13	4.37	5.50	2.00	4.67
2	OV	1.129 × 10^3^	2.656 × 10^3^	2.932 × 10^3^	1.239 × 10^3^	1.963 × 10^3^	3.164 × 10^3^	1.229 × 10^3^	1.460 × 10^3^	1.344 × 10^3^	1.468 × 10^3^
	MV	1.582 × 10^3^	3.775 × 10^3^	3.744 × 10^3^	1.648 × 10^3^	2.691 × 10^3^	4.058 × 10^3^	2.131 × 10^3^	2.349 × 10^3^	2.168 × 10^3^	2.099 × 10^3^
	STD	3.324 × 10^2^	5.237 × 10^2^	4.376 × 10^2^	1.920 × 10^2^	3.985 × 10^2^	4.005 × 10^2^	4.361 × 10^2^	4.966 × 10^2^	4.102 × 10^2^	2.958 × 10^2^
	*p*-value	\	1.510 × 10^−11^ +	1.510 × 10^−11^ +	2.010 × 10^−1^ =	8.066 × 10^−11^ +	1.510 × 10^−11^ +	3.368 × 10^−6^ +	6.270 × 10^−8^ +	8.031 × 10^−7^ +	4.767 × 10^−7^ +
	MR	1.87	8.87	8.83	2.03	6.40	9.17	4.17	5.17	4.47	4.03
3	OV	7.295 × 10^2^	8.088 × 10^2^	8.196 × 10^2^	7.228 × 10^2^	7.554 × 10^2^	8.349 × 10^2^	7.390 × 10^2^	7.373 × 10^2^	7.250 × 10^2^	7.378 × 10^2^
	MV	7.478 × 10^2^	8.337 × 10^2^	8.688 × 10^2^	7.410 × 10^2^	7.905 × 10^2^	8.646 × 10^2^	7.654 × 10^2^	7.729 × 10^2^	7.350 × 10^2^	7.531 × 10^2^
	STD	9.593	1.957 × 10^1^	2.656 × 10^1^	9.491	1.941 × 10^1^	1.869 × 10^1^	1.869 × 10^1^	1.928 × 10^1^	6.905	1.056 × 10^1^
	*p*-value	\	1.510 × 10^−11^ +	1.510 × 10^−11^ +	9.964 × 10^−1^ -	6.028 × 10^−11^ +	1.510 × 10^−11^ +	4.073 × 10^−5^ +	1.014 × 10^−7^ +	1.000 -	1.858 × 10^−1^ =
	MR	3.30	8.17	9.37	2.40	6.53	9.43	4.93	5.50	1.50	3.87
4	OV	1.900 × 10^3^	1.900 × 10^3^	1.900 × 10^3^	1.900 × 10^3^	1.900 × 10^3^	1.900 × 10^3^	1.901 × 10^3^	1.900 × 10^3^	1.901 × 10^3^	1.901 × 10^3^
	MV	1.900 × 10^3^	1.909 × 10^3^	1.900 × 10^3^	1.900 × 10^3^	1.900 × 10^3^	1.900 × 10^3^	1.903 × 10^3^	1.900 × 10^3^	1.902 × 10^3^	1.902 × 10^3^
	STD	0.000	8.282 × 10^−1^	0.000	0.000	0.000	0.000	1.096	0.000	7.668 × 10^−1^	1.036
	*p*-value	\	9.729 × 10^−10^ +	NaN =	NaN =	NaN =	NaN =	6.059 × 10^−13^ +	NaN =	6.059 × 10^−13^ +	6.059 × 10^−13^ +
	MR	3.60	6.70	3.60	3.60	3.60	3.60	9.43	3.60	8.77	8.50
5	OV	1.749 × 10^3^	7.159 × 10^4^	1.941 × 10^5^	7.315 × 10^4^	2.589 × 10^3^	2.201 × 10^4^	4.154 × 10^3^	1.490 × 10^4^	1.930 × 10^3^	1.350 × 10^4^
	MV	2.089 × 10^3^	7.658 × 10^5^	9.379 × 10^5^	1.982 × 10^5^	6.895 × 10^3^	2.672 × 10^5^	6.510 × 10^4^	1.883 × 10^5^	2.243 × 10^3^	9.012 × 10^4^
	STD	1.413 × 10^2^	7.642 × 10^5^	5.684 × 10^5^	1.202 × 10^5^	4.135 × 10^3^	1.758 × 10^5^	5.134 × 10^4^	1.391 × 10^5^	1.522 × 10^2^	7.569 × 10^4^
	*p*-value	\	1.510 × 10^−11^ +	1.510 × 10^−11^ +	1.510 × 10^−11^ +	1.669 × 10^−11^ +	1.510 × 10^−11^ +	1.510 × 10^−11^ +	1.510 × 10^−11^ +	1.510 × 10^−11^ +	1.510 × 10^−11^ +
	MR	1.23	8.73	9.50	6.83	3.07	7.17	4.93	6.40	1.77	5.37
6	OV	1.601 × 10^3^	1.701 × 10^3^	1.923 × 10^3^	1.602 × 10^3^	1.614 × 10^3^	1.628 × 10^3^	1.602 × 10^3^	1.630 × 10^3^	1.603 × 10^3^	1.608 × 10^3^
	MV	1.604 × 10^3^	2.016 × 10^3^	2.414 × 10^3^	1.603 × 10^3^	1.833 × 10^3^	1.769 × 10^3^	1.696 × 10^3^	1.752 × 10^3^	1.605 × 10^3^	1.853 × 10^3^
	STD	5.459	1.826 × 10^2^	2.657 × 10^2^	7.714 × 10^−1^	1.200 × 10^2^	8.822 × 10^1^	9.834 × 10^1^	1.099 × 10^2^	1.022	1.112 × 10^2^
	*p*-value	\	1.510 × 10^−11^ +	1.510 × 10^−11^ +	3.334 × 10^−3^ +	1.845 × 10^−11^ +	1.510 × 10^−11^ +	1.598 × 10^−9^ +	1.510 × 10^−11^ +	2.804 × 10^−5^ +	2.039 × 10^−11^ +
	MR	1.47	8.30	9.90	1.80	6.77	6.10	4.73	5.90	2.87	7.17
7	OV	2.105 × 10^3^	1.299 × 10^4^	1.756 × 10^4^	4.631 × 10^3^	2.746 × 10^3^	1.721 × 10^4^	2.988 × 10^3^	1.479 × 10^4^	2.109 × 10^3^	5.869 × 10^3^
	MV	2.243 × 10^3^	2.191 × 10^5^	7.334 × 10^5^	2.154 × 10^5^	4.161 × 10^3^	1.088 × 10^5^	1.430 × 10^4^	1.599 × 10^5^	2.341 × 10^3^	5.611 × 10^4^
	STD	8.244 × 10^1^	2.547 × 10^5^	5.091 × 10^5^	2.319 × 10^5^	1.746 × 10^3^	6.626 × 10^4^	9.338 × 10^3^	1.387 × 10^5^	9.370 × 10^1^	3.269 × 10^4^
	*p*-value	\	1.510 × 10^−11^ +	1.510 × 10^−11^ +	1.510 × 10^−11^ +	1.510 × 10^−11^ +	1.510 × 10^−11^ +	1.510 × 10^−11^ +	1.510 × 10^−11^ +	1.510 × 10^−11^ +	1.510 × 10^−11^ +
	MR	1.23	7.50	9.30	7.30	3.07	7.23	4.23	7.27	1.77	6.10
8	OV	2.300 × 10^3^	2.349 × 10^3^	2.481 × 10^3^	2.300 × 10^3^	2.300 × 10^3^	2.514 × 10^3^	2.300 × 10^3^	2.300 × 10^3^	2.300 × 10^3^	2.300 × 10^3^
	MV	2.300 × 10^3^	3.521 × 10^3^	3.437 × 10^3^	2.301 × 10^3^	2.301 × 10^3^	3.058 × 10^3^	2.646 × 10^3^	3.421 × 10^3^	2.300 × 10^3^	2.529 × 10^3^
	STD	3.627 × 10^−12^	1.532 × 10^3^	8.239 × 10^2^	7.785 × 10^−1^	1.075	2.608 × 10^2^	8.144 × 10^2^	1.226 × 10^3^	2.670 × 10^−13^	7.235 × 10^2^
	*p*-value	\	1.466 × 10^−11^ +	1.466 × 10^−11^ +	1.466 × 10^−11^ +	1.322 × 10^−2^ +	1.466 × 10^−11^ +	9.645 × 10^−4^ +	1.466 × 10^−11^ +	1.000 -	9.764 × 10^−1^ =
	MR	3.12	8.33	8.60	4.87	4.17	8.17	5.28	7.23	1.03	4.20
9	OV	2.807 × 10^3^	2.910 × 10^3^	3.023 × 10^3^	2.845 × 10^3^	2.832 × 10^3^	2.865 × 10^3^	2.815 × 10^3^	2.825 × 10^3^	2.809 × 10^3^	2.839 × 10^3^
	MV	2.825 × 10^3^	2.960 × 10^3^	3.178 × 10^3^	2.898 × 10^3^	2.868 × 10^3^	2.926 × 10^3^	2.838 × 10^3^	2.855 × 10^3^	2.826 × 10^3^	2.883 × 10^3^
	STD	1.565 × 10^1^	3.079 × 10^1^	7.701 × 10^1^	3.765 × 10^1^	2.503 × 10^1^	2.960 × 10^1^	1.366 × 10^1^	1.826 × 10^1^	1.572 × 10^1^	2.877 × 10^1^
	*p*-value	\	1.510 × 10^−11^ +	1.510 × 10^−11^ +	6.028 × 10^−11^ +	1.749 × 10^−9^ +	1.669 × 10^−11^ +	1.003 × 10^−4^ +	2.766 × 10^−8^ +	6.520 × 10^−1^ =	1.737 × 10^−10^ +
	MR	1.87	8.63	10.00	6.37	5.17	7.83	2.97	4.37	2.03	5.77
10	OV	2.910 × 10^3^	2.931 × 10^3^	3.269 × 10^3^	2.911 × 10^3^	2.900 × 10^3^	2.912 × 10^3^	2.914 × 10^3^	2.913 × 10^3^	2.906 × 10^3^	2.903 × 10^3^
	MV	2.921 × 10^3^	3.000 × 10^3^	3.517 × 10^3^	2.951 × 10^3^	2.962 × 10^3^	2.948 × 10^3^	2.947 × 10^3^	2.918 × 10^3^	2.906 × 10^3^	2.950 × 10^3^
	STD	1.842 × 10^1^	3.229 × 10^1^	1.764 × 10^2^	3.654 × 10^1^	3.320 × 10^1^	3.600 × 10^1^	3.346 × 10^1^	1.591 × 10^1^	3.000 × 10^−2^	3.513 × 10^1^
	*p*-value	\	8.726 × 10^−11^ +	1.481 × 10^−11^ +	4.109 × 10^−6^ +	2.564 × 10^−7^ +	8.557 × 10^−7^ +	1.609 × 10^−7^ +	2.706 × 10^−6^ +	1.000 -	6.854 × 10^−7^ +
	MR	2.83	8.33	10.00	5.67	6.40	5.90	5.03	4.00	1.07	5.77
+/=/-		\	10/0/0	9/1/0	7/2/1	9/1/0	9/1/0	10/0/0	9/1/0	6/1/3	8/2/0
Mean MR		2.15	7.41	8.91	4.69	4.96	7.23	5.01	5.49	2.73	5.55
Final Rank		1	9	10	3	4	8	5	6	2	7

**Table 5:** Statistical results for three simulated maps in robustness experiments.

Scene		Sh = 5	Sh = 10	Sh = 15	Sh = 20
Map1	Sr = 5	7.24 ± 3.13	1.40 × 10^1^ ± 3.65	1.03 × 10^4^ ± 3.80 × 10^2^	1.86 × 10^4^ ± 5.37 × 10^3^
	Sr = 10	1.32 × 10^1^ ± 4.45	1.37 × 10^1^ ± 2.73	1.22 × 10^4^ ± 3.58 × 10^1^	2.25 × 10^4^ ± 6.35 × 10^3^
	Sr = 15	7.28 ± 2.80	1.49 × 10^1^ ± 2.83	1.26 × 10^4^ ± 4.05 × 10^1^	1.97 × 10^4^ ± 5.22 × 10^3^
	Sr = 20	7.44 ± 4.21	1.09 × 10^1^ ± 2.15	1.19 × 10^4^ ± 5.91 × 10^1^	2.05 × 10^4^ ± 4.94 × 10^3^
Map2	Sr = 5	1.38 × 10^1^ ± 1.16 × 10^1^	1.89 × 10^1^ ± 3.18	8.61 × 10^3^ ± 4.01 × 10^3^	2.17 × 10^4^ ± 4.69
	Sr = 10	1.23 × 10^1^ ± 7.25	1.68 × 10^1^ ± 5.09	9.31 × 10^3^ ± 4.23 × 10^3^	2.19 × 10^4^ ± 6.90 × 10^2^
	Sr = 15	1.30 × 10^1^ ± 8.37	1.58 × 10^1^ ± 6.25	1.03 × 10^4^ ± 4.15 × 10^3^	2.18 × 10^4^ ± 2.88 × 10^2^
	Sr = 20	1.09 × 10^1^ ± 1.20 × 10^1^	1.71 × 10^1^ ± 2.90	9.80 × 10^3^ ± 4.28 × 10^3^	2.17 × 10^4^ ± 1.36 × 10^1^
Map3	Sr = 5	4.45 × 10^2^ ± 2.14 × 10^2^	1.22 × 10^3^ ± 3.74 × 10^2^	2.64 × 10^4^ ± 8.95 × 10^3^	5.02 × 10^4^ ± 6.70 × 10^3^
	Sr = 10	5.00 × 10^2^ ± 1.60 × 10^2^	1.24 × 10^3^ ± 6.61 × 10^2^	3.00 × 10^4^ ± 7.92 × 10^3^	5.02 × 10^4^ ± 4.56 × 10^3^
	Sr = 15	5.60 × 10^2^ ± 1.82 × 10^2^	1.48 × 10^3^ ± 6.73 × 10^2^	2.51 × 10^4^ ± 8.51 × 10^3^	5.11 × 10^4^ ± 5.44 × 10^3^
	Sr = 20	6.20 × 10^2^ ± 3.54 × 10^2^	1.76 × 10^3^ ± 1.30 × 10^3^	2.82 × 10^4^ ± 8.69 × 10^3^	5.15 × 10^4^ ± 5.04 × 10^3^

**Table 6:** Statistical results for three simulated maps.

Map	IETO	ETO	HOA	BSLO	GBO	FATA	SAOMPSO	LSMA	L-SHADE	HFPSO
1	5.23 × 10^1^	1.13 × 10^2^	3.32 × 10^2^	7.69 × 10^1^	7.74 × 10^1^	5.07 × 10^3^	6.28 × 10^1^	9.16 × 10^1^	6.58 × 10^1^	9.28 × 10^1^
2	1.82 × 10^1^	5.24 × 10^1^	5.29 × 10^1^	6.49 × 10^1^	4.26 × 10^1^	1.02 × 10^7^	2.52 × 10^1^	2.35 × 10^1^	1.64 × 10^1^	3.83 × 10^1^
3	3.98 × 10^1^	5.22 × 10^2^	5.72 × 10^2^	4.46 × 10^2^	4.93 × 10^1^	1.02 × 10^2^	5.38 × 10^1^	4.33 × 10^1^	4.38 × 10^1^	1.07 × 10^3^

## Data Availability

The data are provided within the manuscript.
